# The National Dental Association of the United States of America

**Published:** 1881-09

**Authors:** 


THE NATIONAL DENTAL ASSOCIATION OF THE UNITED STATES OF AMERICA opened its second annual meeting in Republican Hall, this city, at the time appointed (viz., Aug. 8th, 1881, 11 A. M.).

The meeting was called to order by its President, Dr. A. L. Northrop.

The regular order of business was entered upon by a prayer by Dr. John Allen, calling the roll and payment of dues and reading the record of the last meeting by the Secretary. After some further business of a purely routine nature, in relation to the organization, the morning session closed at sharp one o'clock.

### AFTERNOON SESSION.

After the reports of committees had been received and acted upon the 7th order of business was opened by the reading of a paper by Dr. Frank M. Odell, of New York, followed (as the paper was not complete) by an address, in which the doctor shows the close relation between many so-called diseases, and gave some indications for their treatment. The title of the paper being "Lithiasis."

A paper on "Dental Education" was then read by Dr. M. L. Rhein, of New York. Considerable discussion followed the reading of the papers and after some miscellaneous business, the meeting adjourned at six o'clock.

### SECOND DAY—MORNING.

Following the reading of the minutes, the Association proceeded to the election of officers, resulting as follows :

| | | |
|---|---|---|
| President, | John B. Rich, | New York. |
| 1st Vice-President, | J. B. Patrick, Sr., | Charleston, S. C. |
| 2d Vice-President, | J. H. Smith, | New Haven, Conn. |
| 3d Vice-President, | W. H. Dwinell, | New York, N. Y. |
| 4th Vice-President, | J. R. Walker, | New Orleans, La. |
| 5th Vice-President, | F. A. Levy, | Orange, N. J. |
| Secretary, | R. Finley Hunt, | Washington, D. C. |
| Asst-Sec'y, | Frank M. Odell, | New York. |
| Treasurer, | H. B. Noble, | Washington, D. C. |
| Asst-Treas'r, | John Allen, | New York. |

The Association amended its name by prefixing the word "National," making its present title "The National Dental Association of the United States of America."

A paper was read by Dr. J. B. Patrick, Sr., of Charleston, S. C., on a singular instance of interstitial growth of a tooth.

It was decided that the quadrennial meeting to be held in Washington City next year shall commence on the Thursday after the first Tuesday in August, 1882.

Adjourned to 3 P. M.

At the afternoon session Dr. J. R. Walker, of New Orleans, read a paper on the "Culture of the Teeth," which was extensively discussed.

The President appointed as Executive Committee, Drs. J. Curtiss Smithe and J. B. Ten Eyck, of Washington, and T. S. Waters, of Baltimore.

The Association then adjourned.

The Gala Day of the National Dental Association.—The day following the adjournment of this body the dentists of the city of New York secured the steamer City of Richmond to convey the delegates and their friends to and from Long Branch. At 11 A. M. the happy party (we being an invited guest) steamed out of the harbor amid crafts of all sizes and from all nations, passing Sandy Hook, into the ocean, down the New Jersey coast, landing at the Iron Pier, Long Branch, in due time. A large number of the party donned bathing suits, then for a rough-and-tumble in the surf, a new page in the history of many. The bathing was good.

We have no space to talk about Long Branch, its hotels, private cottages and beautiful drives of more than seven miles along the beach.

Having enjoyed the bathing and several hours on shore, we find ourselves on board and in possession of the steamer's dining room, capable of enjoying the sumptuous dinner provided by our New York brethren. During the repast toasts were drank, &c.

After making the inner man happy, the grand old ocean, with the Queen of Night coming up from its eastern edge, and Sandy Hook light-house on our left, Coney Island and its tower of electric light, surrounded by thousands of lower and lesser ones on our right, made a grand and beautiful picture. With body and soul feasted, after three days of interchange of thought, we find ourselves saying good bye with the renewed hope and brotherly wishes of our college commencement day.

